# Design and Operation of the Transformed National Healthy Start Evaluation

**DOI:** 10.1007/s10995-017-2381-1

**Published:** 2017-12-05

**Authors:** Jamelle E. Banks, Maura Dwyer, Ashley Hirai, Reem M. Ghandour, Hani K. Atrash

**Affiliations:** 1 Division of Epidemiology, Office of Epidemiology and Research, Maternal and Child Health Bureau, Health Resources and Services Administration, 5600 Fishers Lane, Rm 18N118, Rockville, MD 20857 USA; 2 Office of Epidemiology and Research, Maternal and Child Health Bureau, Health Resources and Services Administration, 5600 Fishers Lane, Room 18N122, Rockville, MD 20857 USA; 3 Office of Epidemiology and Research, Maternal and Child Health Bureau, Health Resources and Services Administration, 5600 Fishers Lane, Room 18N120, Rockville, MD 20857 USA; 4 Division of Epidemiology, Office of Epidemiology and Research, Maternal and Child Health Bureau, Health Resources and Services Administration, 5600 Fishers Lane, Room 18N39, Rockville, MD 20857 USA; 5 Division of Healthy Start and Perinatal Services, Maternal and Child Health Bureau, Health Resources and Services Administration, 5600 Fishers Lane, Room 18N29, Rockville, MD 20857 USA

**Keywords:** Healthy start, Evaluation, Infant mortality, Disparities

## Abstract

*Purpose* Improving pregnancy outcomes for women and children is one of the nation’s top priorities. The Healthy Start (HS) program was created to address factors that contribute to high infant mortality rates (IMRs) and persistent disparities in IMRs. The program began in 1991 and was transformed in 2014 to apply lessons from emerging research, past evaluation findings, and expert recommendations. To understand the implementation and impact of the transformed program, there is a need for a robust and comprehensive evaluation. *Description* The national HS evaluation will include an implementation evaluation, which will describe program components that affect outcomes; a utilization evaluation, which will examine the characteristics of women and infants who did and did not utilize the program; and an outcome evaluation, which will assess the program’s effectiveness with regard to producing expected outcomes among the target population. Data sources include the National HS Program Survey, a HS participant survey, and individual-level program data linked to vital records and the Pregnancy Risk Assessment Monitoring System (PRAMS) survey. *Assessment* Descriptive analyses will be used to examine differences in risk profiles between participants and non-participants, as well as to calculate penetration rates for high-risk women in respective service areas. Multivariable analyses will be used to determine the impact of the program on key outcomes and will explore variation by dose, type of services received, and grantee characteristics. *Conclusion* Evaluation findings are expected to inform program decisions and direction, including identification of effective program components that can be spread and scaled.

## Significance

The HS program, which began in 1991, was transformed in 2014. To understand the implementation, utilization and overall impact of the transformed HS program, a national program evaluation is being conducted. This evaluation strives to address the challenges and limitations noted in prior evaluations, most importantly, the lack of an appropriate comparison group, which is essential to determine program impact.

## Purpose

Improving pregnancy outcomes for women and children is one of the nation’s top priorities. The infant mortality rate (IMR) is a widely used indicator of the nation’s health. In 2013, the U.S. IMR was 5.96 infant deaths per 1000 live births. However, racial-ethnic disparities persist and in the same year, the IMR for infants born to non-Hispanic black mothers was 11.11, more than double the non-Hispanic white IMR of 5.06 (Matthews et al. 2015). The Healthy Start (HS) program was created to address factors that contribute to the high IMR, particularly among African-American and other minority groups. The program began in 1991 as a demonstration project with 15 grantees and has expanded over the past two decades to 100 grantees in 37 states and Washington, DC.

The HS program was transformed in 2014 to apply lessons from emerging research, past evaluation findings, and to act on national recommendations from the Report of the Secretary’s Advisory Committee on Infant Mortality (2013). Thus, the HS approach for FY 2014 and beyond builds on the established program infrastructure focusing on individual and family health and adds greater focus on evidence-based practices, standardized approaches, and quality improvement. Grantees are also expected to have an increased role in driving community change, accountability and collective impact. The recommendations re-emphasize the importance of improving women’s health before, during and after pregnancy as a means to improving perinatal outcomes and reducing infant mortality. Further, HS plays an important role in strengthening families and creating the foundation for optimal health and development.

The goal of the transformed HS program is to improve maternal and infant health and to reduce disparities in adverse perinatal outcomes in the US through evidence-based practices, community collaboration, organizational performance monitoring, and quality improvement. To achieve this goal, the HS program employs five community-based approaches to service delivery and facilitates access to comprehensive health and social services for high-risk pregnant women, infants, children through their first 2 years, and their families in geographically, racially, ethnically, and linguistically diverse low-income communities with exceptionally high rates of infant mortality. The five approaches include: (1) improve women’s health; (2) promote quality service; (3) strengthen family resilience; (4) achieve collective impact; and (5) increase accountability through quality improvement, performance monitoring, and evaluation.

Each HS grantee is required to address the five approaches, although they may engage in a diversity of activities within the five approaches. The evaluation has been designed to account for the diversity of HS grantees and activities and will involve documentation of the implementation of the transformed HS program components (e.g., activities, type of services, intervention models) and their alignment with the five HS approaches. This information will be used to examine factors that help explain effective implementation of the transformed HS program.

**Fig. 1:**
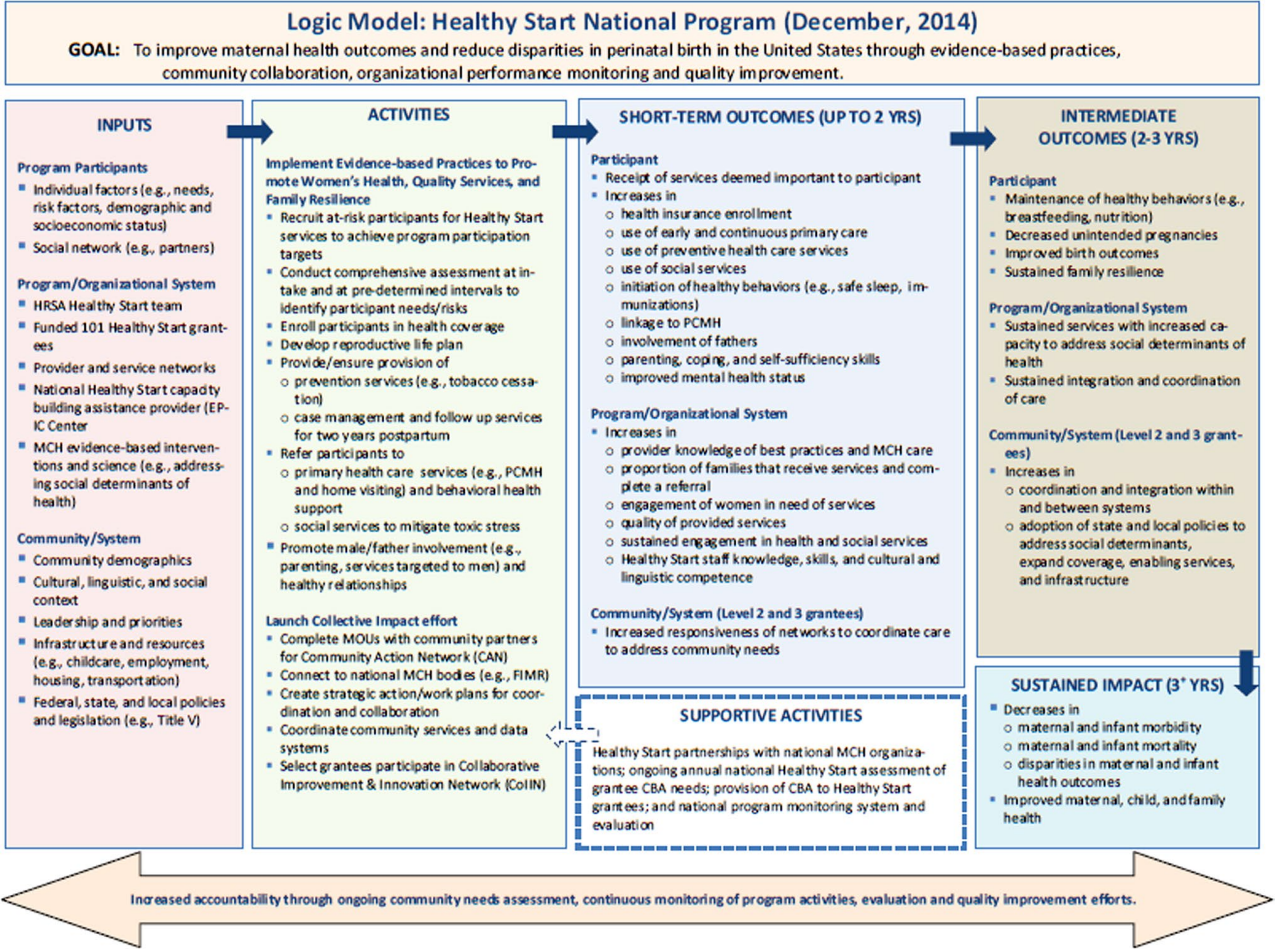
Healthy start national program logic model, December 2014

The five approaches set the framework for the transformed HS program, which is depicted in the program logic model (Fig. 1). The transformed HS program logic model was developed in December 2014 based on the program funding opportunity announcement (FOA). As noted in the program logic model, the HS program relies on a number of resources at the participant, program/organization, and community levels. For example, resources such as social networks and partnerships, provider and service networks, evidence-based interventions and related research, capacity building assistance, community leaders and priorities, community infrastructure and resources (e.g., childcare, housing, transportation) and policies at the Federal, state, and local levels all are essential to the implementation and conduct of HS activities. Implementation of the program’s approaches and subsequent activities is expected to result in a number of outcomes. Short-term outcomes include changes in knowledge, skills, motivation and health care utilization. Intermediate outcomes include changes in healthy behaviors; community, organizational, and systems capacity, quality, efficiency, and effectiveness; and active partnerships and networks. Long-term outcomes are related to changes in health status (for example, morbidity and mortality), policies, and environment.

With the program’s transformation, there is a need to assess its implementation and understand the overall impact of the program using a robust and comprehensive evaluation design. Prior evaluations of HS (Devaney et al. 2000; Brand et al. 2010; Drayton et al. 2015; Health Resources and Services Administration 2006; Howell and Yemane 2006; Rosenbach et al. 2010) demonstrated some positive program impact on access to services, integration of services, maternal health care utilization, knowledge, and behaviors, as well as high participant satisfaction with the HS program. However, the evaluations showed mixed evidence with respect to an association with improved longer-term perinatal outcomes, such as rates of infant mortality, preterm birth, low birthweight and very low birthweight. These evaluations were limited by data quality issues, including inconsistency in the definition and source(s) of some measures; lack of verification of some measures; and missing and incomplete data. Further, the lack of a matched individual comparison analysis prevented strong inference regarding the impact of HS participation on perinatal outcomes.

The Maternal and Child Health Bureau (MCHB) sought the input of an external committee to guide the design and implementation of the national HS evaluation. In October 2014, a Technical Expert Panel (TEP) of maternal and child health researchers, practitioners, project directors and policy stakeholders was convened to discuss and recommend an evaluation design for the transformed HS program. The TEP will serve as an external consultative committee and provide direction on the design and implementation of the evaluation. The evaluation management team will meet quarterly with the TEP to continue to obtain their input and recommendations for the evaluation design, progress and findings, and the final report.

The overarching goal of the national HS evaluation is to determine the effect of the transformed program on participant-level characteristics (e.g. health services utilization, preventive behaviors, and health outcomes). It includes three components: (1) implementation; (2) utilization; and (3) outcome. The purpose of the implementation evaluation is to describe HS programs and strategies and to identify program factors that are associated with effective implementation. The purpose of the utilization evaluation is to examine the characteristics of participants and non-participants and factors that help explain differential penetration, or service rates. The purpose of the outcome evaluation is to assess the overall effectiveness of the program with regard to producing expected outcomes among the target population and factors that help explain variation in the program’s impact on individual level outcomes. The outcome evaluation will employ propensity-score methods, which will include two types of comparisons:

1. A matched individual comparison analysis of linked vital records for HS participants and non-participants in the same general geographic service area for all 100 HS grantees, which maximizes generalizability and will allow for assessment of the key outcome of interest, infant mortality, with adequate statistical power.
2. A matched individual comparison analysis of HS participants and non-participants by oversampling of the Pregnancy Risk Assessment Monitoring System (PRAMS) survey for a random sample of 15 HS grantees. This component of the evaluation data collection strategy will maximize internal validity with a broader set of outcomes and control for matching characteristics that can influence selection into the program.

## Description

**Fig. 2:**
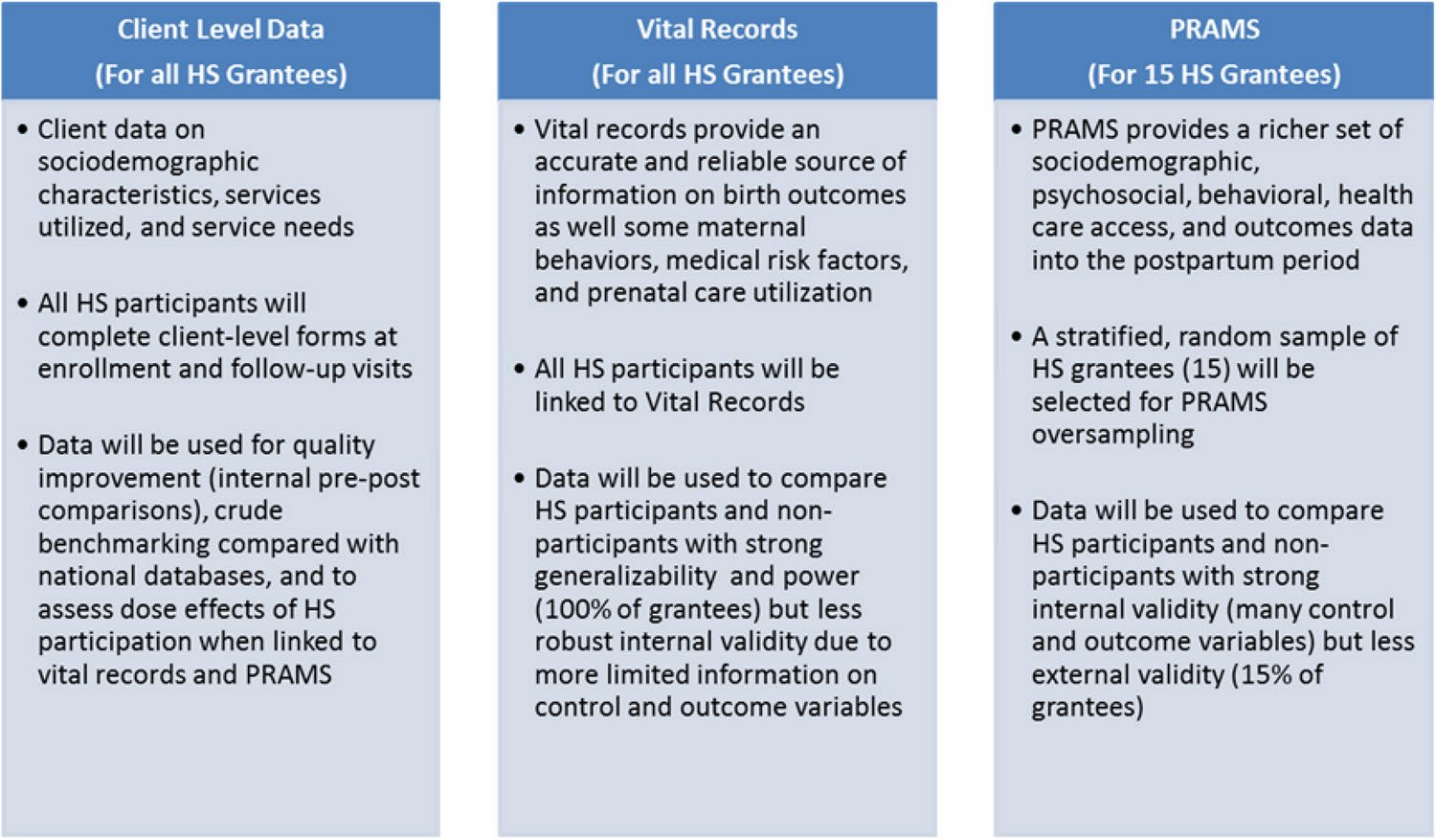
Outcome evaluation data sources

**Fig. 3:**
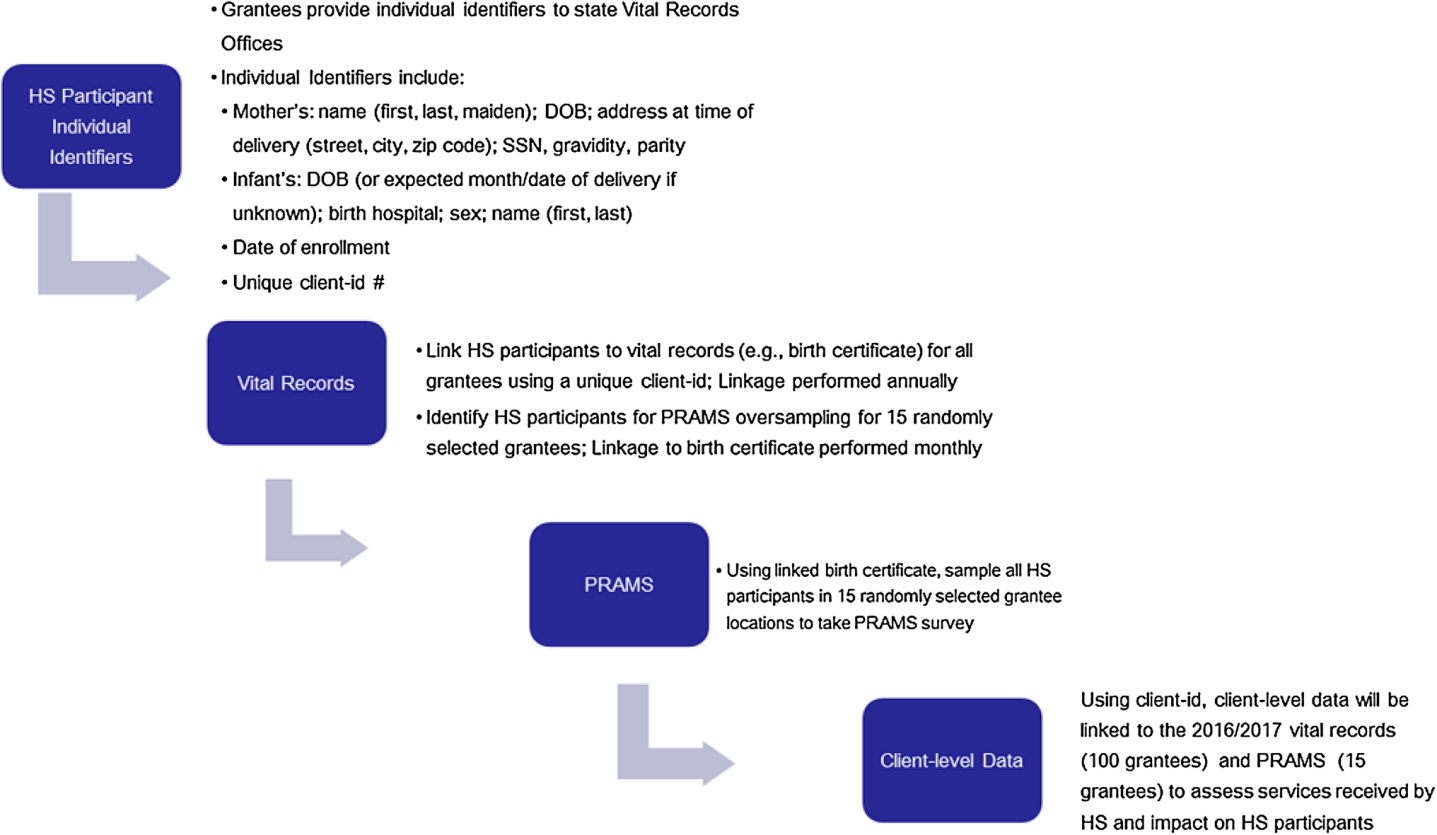
Outcome evaluation flow chart

The primary data source for the implementation evaluation is the National Healthy Start Program Survey (NHSPS). The utilization and outcome evaluations will link state/jurisdiction vital records (e.g., infant birth and death certificates), the Centers for Disease Control and Prevention (CDC) PRAMS survey, and participant-level program data to compare HS participant and non-participant characteristics and outcomes (Fig. 2). HS participant data will be linked to vital records for all HS grantees and to PRAMS data for 15 randomly selected HS grantees, as illustrated in Fig. 3. Key benchmarks and outcomes that can be examined with vital records include infant mortality, low birth weight, preterm birth, initiation and adequacy of prenatal care, breastfeeding initiation, and gestational weight gain. PRAMS provides additional and enhanced data on psychosocial and demographic characteristics, health behaviors and outcomes, and health care access into the postpartum period (see evaluation measures by data source in Table 1).

**Table 1:** Evaluation metrics by data source

	Vitals	PRAMS core Phase 8	Participant Level HS data	HSPS	Other
Benchmarks					
Health insurance (preconception, pregnancy, postpartum)	Partial	X	X		
Well woman visit (preconception)		X	X	Track	NHIS; BRFSS
Postpartum visit		X	X	X	HEDIS
Safe sleep behaviors		X	X		
Ever breastfed	X	X	X	X	NIS
Cigarette smoking (preconception, pregnancy, postpartum)	Partial	X	X		
Interpregnancy interval < 18 months	X	X	X		
Well child visits		X	X	Track	HEDIS
Perinatal depression screening (preconception, pregnancy, postpartum)		X	X		
Intimate partner violence screening (preconception, pregnancy)		X	X		
Additional outcomes and/or characteristics					
Infant mortality	X			X	
Low birth weight	X		X	X	
Preterm birth	X		X	X	
Current breastfeeding		X		Track	
Initiation of prenatal care	X	X	X	Track	
Adequacy of prenatal care	X				
Gestational weight gain	X	X	X	Track	
Weight management counseling (preconception, pregnancy, postpartum)		X	X		
Alcohol use screening		X	X		
Physical activity (preconception, pregnancy, postpartum)		X			
Maternal morbidity	X				
Pregnancy-related complications	X	X	X		
Cesarean section among low-risk first births	X				
Home visiting		X			
Screening or counseling for breastfeeding (pregnancy and postpartum)		X	X		
Screening or counseling for birth control (preconception, pregnancy, and postpartum)		X	X		
Screening for smoking (preconception, pregnancy, postpartum)		X	X		
Screening for drug use (pregnancy)		X	X		
Flu shot receipt and counseling		X	X	Track	
Dental visit		X	X		
Content of postpartum visit		X			
Benchmarks not covered by PRAMS-core or VITALS					
Breastfed at 6 months		Partial	X	X	NIS
Medical home			X		NSCH; DGIS
Follow-up services for perinatal depression			X		
Read daily to child			X		NSCH
Documented reproductive life plan			X	X	
Father and/or partner involvement during pregnancy			X		
Father and/or partner involvement with child 0–24 months			X		
Fully implemented CAN				X	
At least 25% HS participant membership on their CAN membership			X		
QI and performance monitoring process				X	
Fully implemented COIIN process				X	
Healthy start case management dosage					
Duration of enrollment (HS admit date, delivery date, discharge date)			X		
Breadth of interventions—visit type: phone, home, office, other			X		
Amount of contact time—date of visit			X		
HS provider (RN, SW, MH counselor, paraprofessional)			X		
HS enrollment for a prior pregnancy			X		

*Track* the HS survey asked respondents if these items were tracked, *BRFSS* behavioral risk factor surveillance system, *DGIS* discretionary grant information system, *HEDIS* the healthcare effectiveness data and information set, *NIS* national immunization survey, *NSCH* national survey of children’s health

Benchmarking methods will be used to contextualize HS outcomes by comparing individual level outcomes and personal, clinical and socio-demographic risk factors among HS participants to data available from other sources or benchmarks. An attempt will be made to use a low-income reference group for national sources. HS communities are selected based on demonstrated need and thus, will likely demonstrate poorer outcomes than the average community in the United States. HS programs demonstrating progress toward or in reaching the national average will represent an accomplishment but the benchmarking method is unlikely to allow for attribution of any differences to HS program effects.

To link HS participants to vital records data, all 100 HS grantees will collect individual identifiers from eligible program participants (Table 2). Grantees will provide to state/jurisdiction Vital Records Offices (VROs) the individual identifiers for each pregnant and postpartum HS participant with informed consent. The VROs will complete the linkage of HS participants to birth certificates and send the linked data file to MCHB/HRSA (Health Resources and Services Administration). State/jurisdiction VROs will also provide birth certificate data for non-participant controls from the same city or county(s) served by the HS grantee with geographic identifiers (census tract or zip code). After one year, the VROs will update the linkage of HS participants and controls to include any subsequent infant death certificates and send the linked data file to MCHB/HRSA. VROs will transfer birth certificate data to MCHB/HRSA without personally identifiable information for all linked HS participants and non-participants in the same county/city to facilitate analytic comparison. MCHB/HRSA will use the unique client ID to link the vital records data to client-level data and identify the services received by HS participants.

**Table 2:** Individual identifiers for vital records linkage

Linkage variables
**Mother’s name**
**Mother’s date of birth**
Mother’s address at time of delivery
Mother’s social security number
Mother’s race
Mother’s ethnicity
Mother’s medicaid status
Mother’s gravidity
Mother’s parity
Mother’s date of enrollment
**Mother’s unique client ID #**
**Infant date of birth (or expected month or date of delivery if known)**
Infant birth hospital
Infant sex
Infant name
Infant birthweight

Bold = required elements

The National Association of Public Health Statistics and Information Systems (NAPHSIS) will develop a model data sharing/transfer agreement to be adapted and signed for each HS grantee, VRO, PRAMS program and MCHB. This evaluation will be conducted in accord with prevailing ethical principles and program evaluation standards, and is being reviewed by an Institutional Review Board. All participants will provide informed consent prior to their inclusion in the evaluation study.

In addition to linking HS participants to vital records, the evaluation will also link participants to PRAMS survey data. The HS program currently has 86 grantees located in states that conduct the PRAMS survey. To improve the chances of evaluating an operational HS program early in the grant cycle, the PRAMS oversampling was restricted to continuing grantees (75 of 100 total grantees). Similarly, CDC recommended restricting the sample to grantees in states which currently field the PRAMS survey (n = 40) given the potential lack of capacity in new PRAMS Phase 8 states (up to 61 states/jurisdictions/tribes). Therefore, the HS Sampling Frame for the PRAMS oversampling included 63 of 75 continuing grantees that are located in current PRAMS states.

Based on available funding and CDC support services, it was determined that 15 HS grantees could be selected for PRAMS oversampling. To ensure scientific integrity, the 15 HS grantees were randomly selected within strata determined to be of importance to the program. The strata include cells categorized by Grantee Level (1, 2, 3),^1^ Service Area Focus (Urban, Rural, Border, AI/AN), and Region (Midwest, Northeast, South, West). Within the sampling frame, there were only three grantees located in the Western Region (all Level 1 grantees in NM and OR). Given that most Western HS grantees are Urban (7 of 12), a Western Urban Level-1 grantee was selected with certainty. To ensure geographic representation of the remaining regions, Level-2 and Level-3 grantees were selected in the general proportion of these grantees by region. The strata or categories for each level and the methodology for randomly selecting the 15 HS grantee sites can be found in Table 3.

**Table 3:** Strata and methodology for randomly selecting 15 HS grantees sites for PRAMS oversampling

Strata/categories
Select 1 of 2 border grantees (both Level 1)
Select 1 of 3 AI/AN grantees (mostly Level 1)
Select 3 of 28 Level-1 grantees (serving 500+ clients per year)
2 of 19 urban grantees
1 of 18 non-western grantees
1 of 1 western grantees
1 of 9 rural grantees
Select 5 of 17 Level-2 grantees (serving 800+ clients per year)
4 of 14 urban grantees
2 of 5 midwestern grantees
1 of 4 northeastern grantees
1 of 5 Southern grantees
1 of 3 rural grantees (all South)
Select 5 of 13 Level-3 grantees (serving 1000+ clients per year; all urban)
1 of 2 midwestern grantees
2 of 6 northeastern grantees
2 of 5 Southern grantees
Methodology for randomly selecting 15 grantee sites within each stratum
Grantee lists were entered into different excel spreadsheets by the five primary strata listed above
Within each stratum-specific spreadsheet, each grantee is given a random number between 0 and 1 (formula “=RAND()”), with values copied and pasted so that numbers do not regenerate
The spreadsheets were sorted by random number (lowest to highest) and the top-listed grantees were selected according to primary and secondary stratum-specific criteria listed above
Any grantee/state that refuses participation will be replaced by the next listed grantee/state within the particular stratum

Beginning in 2017, 15 randomly selected HS grantees will send individual identifiers (Table 2) for pregnant and postpartum HS participants to their state/jurisdiction VROs. The VROs will link to the birth certificate and note which individuals are HS participants. PRAMS offices in the randomly selected states will sample these individuals to take part in the PRAMS survey (2–9 months postpartum). Oversampling via PRAMS will require ongoing monthly linkage to identify HS participants for monthly batch sampling. The CDC will provide MCHB with the full PRAMS file of all PRAMS participants in the selected states (both HS participants and non-participants), including linked vital records and geographic identifiers for analytic purposes. State/jurisdiction VROs will then transfer any subsequent infant death certificate data for the PRAMS sample to MCHB. Finally, MCHB will link client-level program information on service receipt within HS to PRAMS and vital records data, using the unique client ID number, to complete evaluation analyses. This will allow the evaluation team to fully assess the type, dose and frequency of services HS participants received and the impact these services had on important benchmark and outcome measures.

Individual-level matching will ensure that the comparisons in the evaluation involve similar women (with the exception that the participants have accessed the transformed HS program), and the evaluation produces estimates of the effects of HS on individual-level outcomes. A propensity score matching approach will be used to match participants and non-participants. The propensity score method uses the set of variables to compute the probability of being served by HS for each HS participant and nonparticipant using a logistic regression model. In other words, the demographic and risk factors (independent variables) are used to predict whether individuals are HS participants (dependent variable). The resulting propensity scores are the chances that each individual is a HS participant, or the predicted propensity to be a HS participant.

Given the general PRAMS sample size, however, non-participants will not be restricted to the same “community” in the PRAMS matched comparison. Thus, census tract or zip code-based poverty will likely be used to control for community characteristics. In the vital records comparison, however, participants will be able to be matched to non-participants in the same general geographic area (city/county) that is served by HS (e.g., same census tract service area or a census tract in the same city with similar rates of disadvantage as those served by HS). This will enhance MCHB/HRSA’s ability to draw conclusions about the effectiveness of the program in influencing the key health outcomes of the transformed program. Given that there are likely to be many more non-HS participants in vital records than HS participants, the analysis could be statistically strengthened by a 1:N (3, 4) match.

## Assessment

The implementation evaluation will use program and participant survey data to develop metrics to assess more (versus less) effective implementation of HS services. Program goals and fidelity to implementing the five HS approaches will be analyzed by assessing, for example, number of participants enrolled in health coverage and methods used for enrollment, use of standardized curricula and interventions across grantee sites, and types of prevention education models used. We aim to identify program approaches/models considered to be key to effective implementation and identify metrics for assessing performance.

The analysis will also test the statistical significance of bivariate and multivariable associations between program- and organization-level factors and indicator(s) of effective implementation. Program-level factors may include the outreach strategies employed; number and types of referrals provided; the number and types of screenings provided; case management models utilized; caseloads maintained; and promotion of male involvement, among others. Organization-level factors will likely include the type of program (urban, rural, border); the HS program level (1, 2 or 3); the lead agency type; age of the program; staffing characteristics; and the type of approaches and services provided, among others.

Analysis of the utilization evaluation will include descriptive analyses of HS participants in terms of a number of individual characteristics, including socio-demographic indicators, health behaviors, utilization of non-HS health services and health outcomes. Bivariate analyses will test for statistically significant differences in health behaviors, health service utilization patterns, and health outcomes between HS and non-HS participants and among HS participants, by level of utilization of HS services. Descriptive analyses will also examine service or penetration rates by intended target characteristics (e.g., % of uninsured or Medicaid-insured served) and summarize utilization levels among participants at the grantee level.

The outcome evaluation analysis will estimate the effect of program participation by comparing outcomes of HS participants and non-participants using multivariable techniques. Individual-level propensity score matching (see examples of matching variables in Table 4) will ensure that outcome comparisons between participants and non-participants are balanced with respect to observed characteristics. Linkage and inclusion of all delivering participants, regardless of participation level, will help to reduce bias due to attrition or loss-to-follow-up. Multiple comparison groups, including internal references among program participants, will be used to test the sensitivity of results and promote causal inference (e.g. postpartum versus prenatal enrollees, dose-response effects). Analyses will also examine variation in effects by program and organizational characteristics to identify critical practices that can be spread and scaled to maximize impact across grantees.

**Table 4:** Examples of matching variables to be included in multivariable models

Variable	Vital records	PRAMS
Age	X	
Race/ethnicity	X	
Parity	X	
Plurality	X	
Education	X	
Marital status	X	
Neighborhood poverty rate	X^a^	
Body mass index	X	
Medical risk factors	X	X
WIC participation	X	X
Health insurance	X	X
Household income		X
Time of PRAMS survey completion		X
Physical abuse (before, during, and after pregnancy)		X
Stressful life events		X
Preconception visit		X
Pregnancy intention		X
Preconception health status		X
Postpartum depression		X

^a^From residential address geocoding

## Conclusion

The national evaluation of the transformed HS program is important for several reasons; it (1) seeks to assess the transformed HS program, which was designed to apply lessons from emerging research, past evaluation findings, and the Secretary’s Advisory Committee on Infant Mortality; (2) addresses the principal limitations of previous HS evaluations by employing two types of matched individual comparison analysis and the use of standardized datasets; and (3) will help further our understanding of the “evaluability” of complex public health interventions. Further, evaluation findings are expected to inform program decisions and future program direction and to enable not only a determination of whether HS is effective in impacting participant outcomes, but why and how, so that effective program components can be spread and scaled.

## Footnotes

1 Level 1 Community-based HS programs serve a minimum of 500 participants per year and implement activities under the five approaches; Level 2 Enhanced Services grantees serve a minimum of 800 participants and engage in Level 1 activities as well as activities to stimulate community collaboration; Level 3 Leadership and Mentoring HS grantees serve a minimum 1000 participants and engage in activities under Levels 1 and 2, as well as activities to expand maternal and women’s health services, develop place-based initiatives, and serve as centers to support other HS and similar programs.
